# The relationship between positive youth development and internet gaming disorder in Chinese adolescents: A moderated mediation model

**DOI:** 10.1371/journal.pone.0276174

**Published:** 2022-11-03

**Authors:** Xiong Gan, Ke-nan Qin, Min Li, Hao Li, Xin Jin, Cheng-fu Yu

**Affiliations:** 1 Department of Psychology, College of Education and Sports Sciences, Yangtze University, Jingzhou, China; 2 Department of Psychology, College of Education and Sports Sciences, Yangtze University College of Technology and Engineering, Jingzhou, China; 3 Department of Psychology and Research Center of Adolescent Psychology and Behavior, School of Education, Guangzhou University, Guangzhou, China; The Hong Kong Polytechnic University, HONG KONG

## Abstract

Internet gaming disorder (IGD) is a social problem that cannot be ignored. Considerable research has shown that IGD can impede the healthy physical and mental development of adolescents. Based on positive youth development theory and stage-environment fit theory, the current study explored the mediating effect of depression and the moderating effect of gender to determine the association between positive youth development (PYD) and IGD. A sample of 1970 Chinese adolescents aged 11–18 years (1021 boys, 940 girls, and 10 unidentifiable individuals) completed questionnaires related to PYD, depression, IGD, and their background information. The results revealed that PYD negatively predicted IGD. After controlling for gender and age, this study found that depression mediated the relationship between PYD and IGD. Furthermore, gender moderated the relationship between depression and IGD. Boys with depressive symptoms were more likely to indulge in IGD than girls. This research contributes to a more thorough understanding of how PYD decreases the risk of IGD. These findings suggest that cultivating PYD attributes is a promising approach to prevent or reduce depression and IGD among adolescents in mainland China.

## Introduction

Hall once described adolescence as a “stormy” time in one’s life [1]. Adolescence is a pivotal transition period when individual physiological development is quite rapid. Adolescents’ fast physiological changes will impact their psychological development. Researchers in the subject of positive development have always been interested in how this stage develops. On the one hand, adolescent problems in psychology and behavior will negatively affect their current studies and lives. On the other hand, these issues will persist throughout maturity, affecting their growth [2]. According to the China Internet Network Information Center’s (CNNIC), the 48th China Statistical Report on Internet Development indicated that there were 1.011 billion internet users in China as of June 2021, with online gaming users accounting for 52.4 percent of the total. Teenagers aged 10 to 19 account for 12.3% of all internet users [3]. As a result, Internet gaming disorder (IGD) among teenagers is a considerable issue affecting their development in China [4]. IGD refers to an individual’s uncontrollable, excessive, and compulsive use of online games that causes social and/or emotional problems [5]. The World Health Organization (WHO) [6] has included “gaming disorder” in the International Classification of Diseases (ICD-11) in 2018, reminding us of the importance of appropriate interventions. A large body of evidence has indicated that IGD has a serious negative impact on adolescent development [7, 8]. For instance, IGD was found to be associated with high hostility, high loneliness, low academic achievement, low social competence, low self-esteem, low self-confidence, low life satisfaction, and low well-being [7, 9, 10]. Besides, excessive use of online games can also disrupt sleep patterns, lead to fatigue, and lower a person’s immune system [5].

Previous research and intervention programs have primarily focused on developmental deficits and the impact of external variables on problem behaviors in adolescents. But this defect-focused model overlooks the benefits that can support teenagers’ development, and it may erroneously classify youth as problematic and inept individuals, leading to more issues [2, 11]. With the growth of positive psychology, researchers are paying greater attention to the positive youth development (PYD) perspective rather than just intervening and treating their harmful behaviors. Many previous studies have single-handedly explored problematic game use in adolescent populations in a Western context [8, 9], but these findings do not always apply to non-Western countries (e.g., China). Unfortunately, only a few studies have looked at Chinese adolescents and investigated the relationship between PYD and addictive behaviors [12, 13], particularly regarding the mechanisms of PYD on IGD. Therefore, considering the prevalence of internet use in Chinese adolescents, the current study aimed to examine the predictive effects of PYD on IGD, the mediating effect of depression, and the moderating effect of gender. It is of practical significance to promote adolescent development positively and to prevent and reduce their depression and IGD.

### Positive youth development and internet gaming disorder

Positive youth development involves a number of theories, including bio-ecological theory, development assets theory, developmental systems model, and so on [14–16]. Some researchers have identified 15 PYD attributes that are commonly emphasized in effective PYD programs [17]. These PYD attributes cover a series of positive internal assets from one’s inner world, as well as positive experiences derived from the external world. In contrast to a problem-centered perspective, the PYD perspective argues that various youth problems, including IGD, can be mitigated and avoided by providing PYD attributes [2]. This perspective emphasizes that thriving happens when adolescent strengths are aligned with resources in their contexts, and it focuses on the strengths to be explored rather than the issues to be fixed. Individuals act in their contexts to identify, access, and use resources by constructing an appropriate environment and then achieve positive development by integrating their internal strengths and external support [16]. Theoretically, individuals are active producers of their own development, and youth development is “relatively plastic.” Even teens in adversity will awaken their potential, buffer life stress, and accomplish positive development as long as the external environment provides sufficient developmental assets [18].

A previous study noted that developmental assets are the protective factors for the positive development of adolescents, and the less assets youth possess, the more likely they are to exhibit internalizing and/or externalizing problems (e.g., depression and IGD) and the less likely they are to succeed and demonstrate healthy behaviors [19]. According to stage-environment fit theory [20], changes in the developmental needs of adolescents are closely related to changes in their social contexts. If the developmental assets in their surroundings do not match their inner changing needs during this period, it can have a seriously negative impact on their current and subsequent development. Previous studies have also shown that PYD not only positively predicts individual life satisfaction, well-being, and healthy developmental outcomes (e.g., academic achievement), but also is a negative predictor of various internalizing and externalizing problems and problem behaviors (e.g., internet addiction, social networking addiction, and delinquency) [11–13, 21–24]. Besides, an evaluation of the P.A.T.H.S. program in Hong Kong and a PYD program in Mainland China both found that adolescents can be effectively shaped and achieve healthy development [25, 26]. Given the theories and previous findings above, the present study hypothesizes that PYD would negatively predict adolescent IGD (Hypothesis 1).

### Depression as a mediator

In addition to the direct effect of PYD on IGD in adolescents, our understanding of the involved emotional mechanisms remains unclear. Depression is a prevalent psychological health question among adolescents, and it relates to the poor emotional experience of people who feel powerless to deal with external pressure [27]. Depression can have a variety of severe repercussions for adolescent development, including poor academic achievement [28], anxiety [29], non-suicide self-harm, and suicidal ideation and behavior [30], and the bad impacts on teenagers’ physical and mental health can last into adulthood [31]. Young people with a high degree of development are more likely to have an optimistic attitude, and then they are more able to deal with setbacks or bad situations, modify themselves and respond positively to reduce their depression [25, 32]. Previous evidence has also suggested that PYD attributes are beneficial for protecting adolescents from depression [33–35]. For example, Milot Travers and Mahalik [33] found that lower levels of PYD attributes were significantly linked to a higher prevalence of depression among teenagers. A three-year longitudinal study has revealed that PYD is a negative predictor of future depressive symptoms [34]. Besides, a two-wave design study also indicated that higher PYD attributes predicted a drop in Chinese adolescent depression both concurrently and longitudinally [35]. Therefore, the level of PYD has a vital impact on adolescent depression. Specifically, those with higher levels of positive development had lower rates of depressive symptoms.

Furthermore, the link between depression and addictive behaviors (e.g., internet addiction) is stronger than the link between depression and other maladaptive problems [36]. High levels of depression are associated with increased IGD [37]. Researchers have found that depression is a risk factor for IGD in adolescents [38]. This is because addictive behaviors are often used as strategies to help adolescents escape from realistic outcomes and negative experiences as they adjust to their emotions [39]. According to the psychological decompensation hypothesis [40], internet surfing is a compensatory behavior used by teens when their psychological development is impeded. Depression hinders the normal development of adolescents and prevents them from actively coping with and solving difficulties. Young people are more likely to find fulfillment by immersing themselves in online games, which give them a new identity and create a virtual space where they can temporarily forget about their problems and escape from a dissatisfying reality [41]. The use of the internet has been strengthened as a result of this loss-compensation-satisfaction paradigm, and excessive usage of the internet has ultimately induced the IGD. As a result, depression may positively predict IGD among adolescents. Given the above theoretical and empirical evidence, this study hypothesizes that depression would act as a mediator between PYD and IGD (Hypothesis 2).

### Gender as a moderator

There are gender differences in many aspects of adolescent development, and gender identity is a process of continuous life-long development [42]. So, the mediating model described above may also exhibit gender differences. The multidimensional model of gender identity states that boys and girls take account of group attributes and characteristics, social expectations, and so on when developing their psychology and implementing actions. Eventually, boys’ and girls’ groups socialize different behaviors and social rules [43]. According to previous research [7, 44], gender is an important demographic variable in IGD. For starters, gender disparities in behavioral patterns and traits, motivations for internet usage, and the severity of IGD exist. Male adolescents are more likely than females to play online games, and they are more inclined to put their studies, employment, and even sleep on hold in order to do so [8, 10]. Second, the predictors of IGD were shown to be different for males and females. The empirical findings showed that male adolescents with lower self-esteem and lower satisfaction with daily life were more easily addicted to playing online games, whereas these characteristics had no predictive influence on females [45]. In addition, some scholars have also reported that the connection between depression and internet addiction depends on gender. Depression was found to significantly predict eventual internet addiction in male adolescents, while internet addiction was found to be a substantial predictor of subsequent depression in female adolescents [46].

In view of the above, it is necessary to investigate the gender disparities in the mechanism of IGD in adolescents. Some scholars claimed that IGD had been more generally researched among adolescents who had previously played online games, but whether similar patterns of gender differences would manifest in the same group of adolescents is ambiguous and needs to be studied further [45]. The aim of this study was to examine the moderating role of gender in adolescents for the second half of the above mediated model pathway. As a consequence, we estimate that boys with depression are more susceptible than girls to becoming obsessed with online games and developing IGD (Hypothesis 3).

### The present study

**Fig 1:**
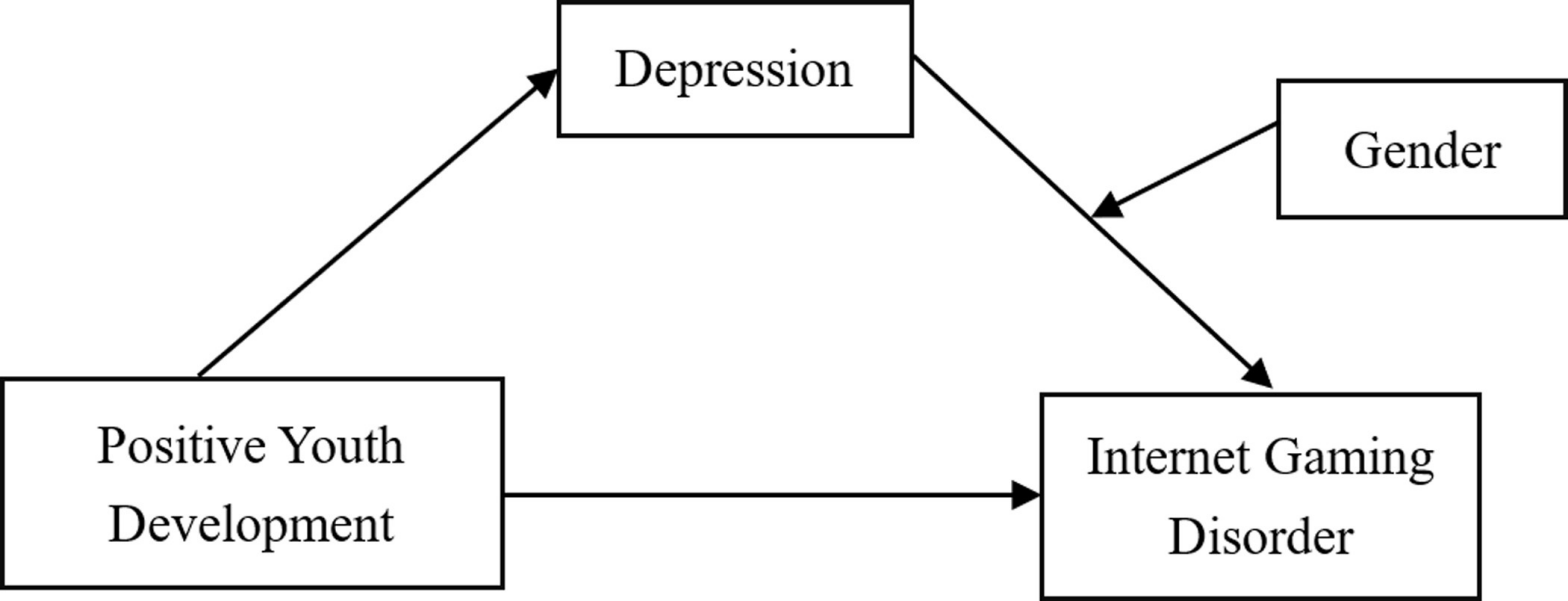
A moderated mediation model of positive youth development, depression, internet gaming disorder and gender.

Despite the numerous previous studies in the field of IGD, there are still some research gaps. First, researchers have mostly focused on the effects of negative factors on adolescent IGD at the expense of positive factors. Second, some single positive factors have been found to be effective in alleviating IGD in adolescents, but there is a lack of research on the effect of overall positive qualities (e.g., PYD) on IGD and internal mechanisms. Third, there are very few studies on the relationship between PYD and IGD in Chinese adolescents. In view of the above, this study established a moderated mediation model based on a Chinese context (as shown in Fig 1), aiming to test the following hypotheses: (1) PYD could negatively predict adolescent IGD; (2) depression could mediate the relationship between PYD and IGD; and (3) gender could moderate the second half of the mediating role of depression.

## Methods

### Participants and procedures

Randomized cluster sampling was used to collect data from students in Grades 7 and 8 at a private school in Jingzhou and Grades 10 and 11 at a public school in Shiyan, Hubei province, mainland China. A total of 2,117 questionnaires were distributed, of which 1970 (93.1%) were valid. Among these participants, 1,021 (51.82%) were boys, 904 (47.72%) were girls, and 10 (0.51%) did not report their gender information. The age of the subjects ranged from 11 to 18 years old (M_age_ = 15.34 years, SD = 1.39 years).

After obtaining informed consent from school administrators, teachers, and participating students and their guardians (parents), trained-well teachers and research assistants explained the purpose of the study to all students and asked them to complete the questionnaire independently. In addition, we also emphasized some vital principles, including confidentiality, anonymity, voluntary participation, do-no-harm, and free withdrawal. In this study, students were asked to complete a pen-and-paper test, taking a class as a unit, in 20 minutes, and all questionnaires were collected immediately thereafter. Besides, this study also received approval from the Ethics Review Committee of Yangtze University.

### Measures

#### Positive youth development

The 90-item “The Chinese Positive Youth Development Scale” (CPYDS) was adopted in this survey. Shek et al. developed a 15-dimensional scale to measure the positive development of Chinese youth [47], including bonding, resilience, social competence, recognition of positive behavior, emotional competence, cognitive competence, moral competence, self-determination, self-efficacy, clear and positive identity, beliefs in the future, prosocial involvement, prosocial norms, and spirituality. All items were on a six-point Likert scale (1 = strongly disagree, 6 = strongly agree), and 17 items were scored in reverse. The mean value (TPYD) of the total scores of 15 dimensions was used to represent the level of positive adolescent development in this study. This scale has been widely used and has demonstrated high reliability and validity in previous studies [23, 48]. The Cronbach’s alpha values of the above-mentioned 15 dimensions were between 0.71 and 0.91, and the Cronbach’s alpha was 0.97 for TPYD in this study.

#### Depression

The Center for Epidemiological Studies Depression Scale (CES-D) is widely used to investigate adolescents, adults, and older adults for depressive symptoms all over the world [49]. Wang et al. translated the revised Chinese version of the CES-D [50], which had a total of 20 items, 4 of which were reverse-scored. In our survey, adolescents were asked to report depression, feelings of worthlessness, helplessness, hopelessness, psychomotor retardation, loss of appetite, sleep disorders, and poor concentration within the last week. Each item was answered on a four-point scale. 0 means “rarely or none of the time (less than 1 day)”, 1 means “some or a little of the time (1 to 2 days)”, 2 means “occasionally or a moderate amount of time (3 to 4 days)”, and 3 means “most of all the time (5 to 7 days)”. The higher the mean score, the higher the level of depression among teenagers. In previous studies, this scale also showed great reliability and validity [34, 51]. In this study, Cronbach’s alpha for the CES-D was 0.90.

#### Internet gaming disorder

Using an 11-item questionnaire invented by Gentile [52], we used a three-point rating scale (0 = never, 2 = frequently) adapted and translated by Yu et al. [53] to assess the condition of IGD in adolescents over the past 6 months (e.g., “Have you ever spent too much time playing internet games and got bad grades?”). The data was recorded (0 = never, 0.5 = sometimes, 1 = frequently). This method distinguishes “sometimes” from “frequently” and takes into account students who have occasionally experienced the symptoms of IGD, so it is more accurate than “whether or not to score” (yes = 1, no = 0) [51]. The higher scores for all the items indicate the greater tendency of IGD, and we calculated the average score for all the items in this study. The earlier results showed that this scale has good reliability and validity in Chinese adolescents [54]. In this study, Cronbach’s alpha was 0.86 for IGD.

### Statistical analysis

Firstly, the present study employed SPSS software (version 25.0) to process the missing data. We used the mode to fill in the missing data for gender and the linear interpolation estimates to deal with the missing data for the other variables. Secondly, our study emphasized the confidentiality of data and controlled the reverse scoring and possible common method biases in the procedure. The Harman single-factor test was used to verify the extent of the bias caused by common method variance [55, 56]. The results showed that there were 21 factors with eigenvalues greater than 1, and the interpretative rate of the first common factor was 26.51% (less than 40%), suggesting that there was no severe common method bias in our study. Thirdly, descriptive statistics and correlational analyses were conducted among key variables in SPSS 25.0. Fourthly, to investigate the mediating role of depression (Model 4) and the moderating mediation model of depression and gender (Model 14) between PYD and IGD, we chose to use the SPSS macro PROCESS. In addition, Preacher and Hayes argued that the significance of mediating and moderating effects was widely used in the bootstrapping method so as to obtain robust standard errors for parameter estimation [57]. This method can produce 95% bias-corrected confidence intervals (CIs) from 5,000 resamples of the data, which is regarded as significant if the CIs do not contain zero. Finally, simple slope analysis was performed to decompose significant interaction effects.

## Results

### Descriptive statistics and correlation analyses

The results of the descriptive statistics (including mean and SD) and correlations among variables are presented in Table 1. In this study, PYD was negatively correlated with depression and IGD. Depression and IGD were positively correlated with each other. Furthermore, the results of the independent samples t-test showed that there was no significant difference in the levels of PYD (M _female_ = 4.70, M _male_ = 4.69, *p* = 0.83) and depression (M _female_ = 0.80, M _male_ = 0.78, *p* = 0.29) between females and males. But the level of IGD of female students was significantly lower than that of males (M _female_ = 1.29, M _male_ = 1.53, *p* < 0.001).

**Table 1:** Descriptive statistics and correlations of the key variables.

Variables	M	SD	1	2	3	4
1.Gender	-	-	-			
2.PYD	4.70	0.69	-0.01	-		
3.Depression	0.79	0.55	-0.02	-0.57***	-	
4.IGD	1.41	0.37	0.32***	-0.30***	0.31***	-

Note. N = 1970. PYD, Positive Youth Development; IGD, Internet Gaming Disorder. Gender was a dummy variable in this model such that 0 = female and 1 = male, the same below.

***p < 0.001.

### Testing for mediation effect

The present study utilized Model 4 in PROCESS to investigate the mediating effect of depression between PYD and IGD, as suggested by Hayes [58]. Based on previous research, we chose to control for gender and age [12, 34] in this study. The relevant results of the regression analyses and the mediating effect analysis are shown in Table 2. To begin, after controlling for covariates, the results revealed that PYD predicted IGD negatively (B = -0.29, *p* < 0.001) (Model 1). Secondly, PYD was a significant predictor of depression (B = -0.57, *p* < 0.001) (Model 2). Third, after adding depression as a mediator, PYD still had a negative effect on IGD (B = -0.17, *p* < 0.001), and higher depression predicted increasing IGD (B = 0.21, *p* < 0.001) (Model 3). Finally, the bias-corrected bootstrapping mediation test showed that the indirect effect from PYD to IGD through depression was significant (B = -0.12, 95%CI = [-0.15, -0.08]). In addition, the mediation effect accounted for 41.38% of the total effect. Consequently, the relationship between PYD and IGD was partially mediated via depression.

**Table 2:** Regression result for the mediation model.

Model							
Model 1: Total effect model (IGD)							
R	R^2^	F	df_1_	df_2_	B	SE	t
0.44	0.20	160.26***	3	1966			
Constant					0.25	0.23	1.10
Gender					0.63	0.04	15.63***
Age					-0.04	0.02	-2.54*
PYD					-0.29	0.02	-14.31***
Model 2: Mediating variable model (Depression)							
R	R^2^	F	df_1_	df_2_	B	SE	t
0.57	0.33	323.42***	3	1966			
Constant					0.29	0.21	1.33
Gender					-0.06	0.04	-1.52
Age					-0.02	0.01	-1.21
PYD					-0.57	0.02	-30.53***
Model 3: Dependent variable model (IGD)							
R	R^2^	F	df_1_	df_2_	B	SE	t
0.48	0.23	143.18***	4	1965			
Constant					0.20	0.23	0.85
Gender					0.65	0.04	16.21***
Age					-0.03	0.01	-2.35*
PYD					-0.17	0.02	-7.12***
Depression					0.21	0.02	8.61***
Mediating effect analysis							
		B	SE	LLCI	ULCI	Ratio of the effect to the total effect	
Total effect		-0.29	0.02	-0.33	-0.25		
Direct effect		-0.17	0.02	-0.22	-0.13	58.62%	
Indirect effect		-0.12	0.02	-0.15	-0.08	41.38%	

Note. N = 1970. Unstandardized regression coefficients are reported. Bootstrap sample size = 5000. CI, confidence interval; LL, lower limit; UL, upper limit.

*p < 0.05.

***p < 0.001.

### Testing for moderated mediation

This study used Model 14 of PROCESS [58] in order to test whether the association between depression and IGD was moderated by gender. The results of the moderated mediation analysis are displayed in Table 3. The interaction of depression and gender significantly and positively predicted adolescent IGD (B = 0.17, *p* < 0.001) (Model 4). In other words, gender moderated the influence of depression on IGD in this study (B = -0.09, 95%CI = [-0.15, -0.04]), indicating that the moderated mediating effect is valid.

**Table 3:** Regression results for the moderated mediation model.

Model							
Model 4: Moderated mediation model (IGD)							
R	R^2^	F	df_1_	df_2_	B	SE	t
0.48	0.23	119.01***	5	1964			
Constant					0.21	0.23	0.91
Age					-0.04	0.01	-2.41*
PYD					-0.17	0.02	-7.05***
Gender					0.65	0.04	16.26***
Depression					0.12	0.03	3.97***
Gender × Depression					0.17	0.04	4.19***
Conditional indirect effect analysis of gender							
				B	SE	LLCI	ULCI
Male				-0.17	0.02	-0.21	-0.12
Female				-0.07	0.02	-0.11	-0.03

Note. N = 1970. Unstandardized regression coefficients are reported. Bootstrap sample size = 5000. CI, confidence interval; LL, lower limit; UL, upper limit.

*p < 0.05.

***p < 0.001.

**Fig 2:**
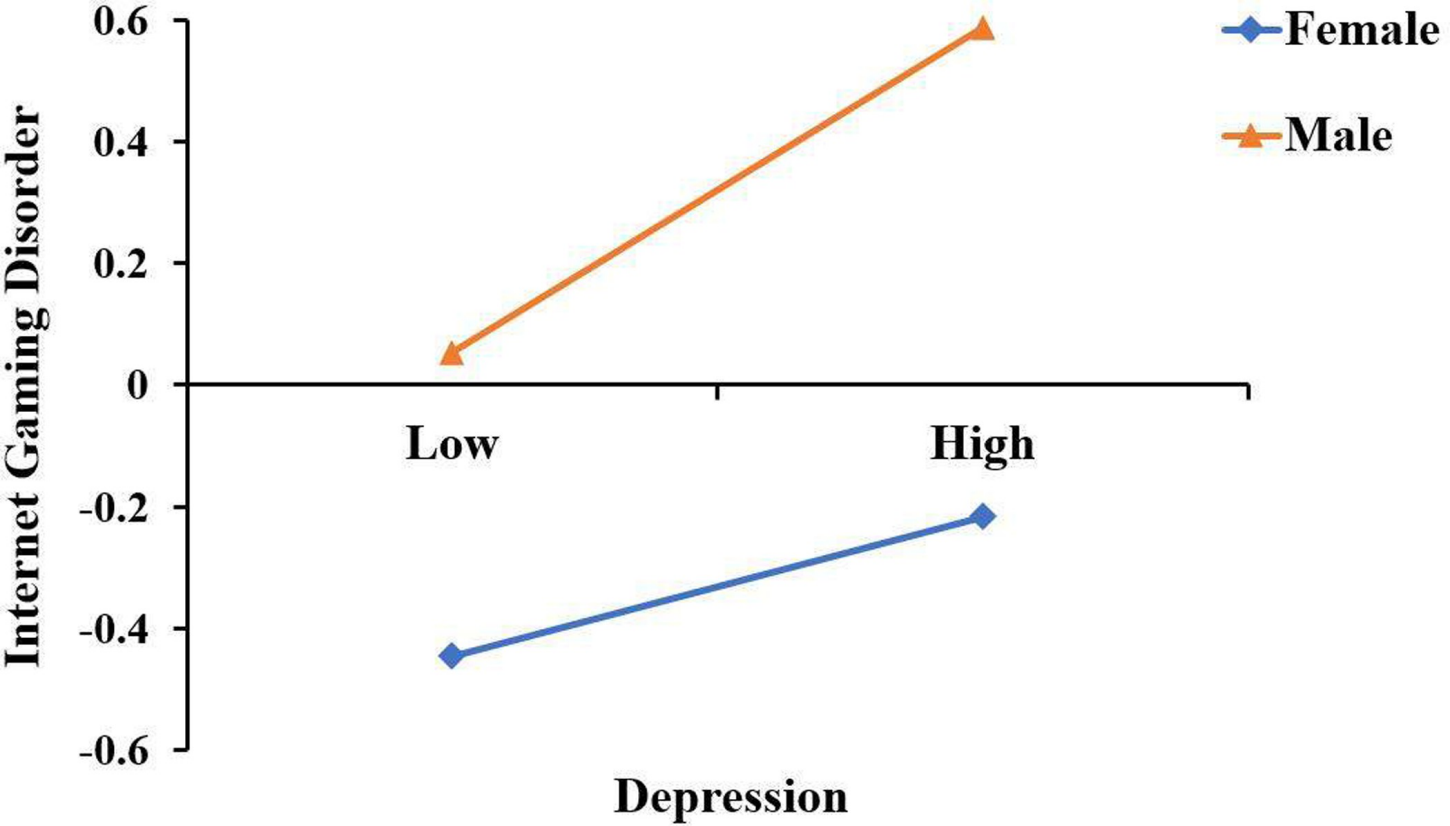
Gender as a moderator in the relationship between depression and IGD.

To further understand the substance of the interaction effect between depression and gender, we analyzed the mediating effect of depression between PYD and IGD among males and females, and related results are presented in Table 3. The result showed that the mediating effect of depression in both boys and girls was significant. As can be perceived from Fig 2, a simple slope test showed that female adolescents with depressive symptoms had a significant predictive effect on IGD (*β*_*simple*_ = 0.12, *p* < 0.001) and males with depressive symptoms had a greater predictive effect on IGD (*β*_*simpl*e_ = 0.29, *p* < 0.001). Consequently, the positive predictive effect of depression on IGD was statistically significant in both boys and girls. And compared with females, male students with depression were more likely to indulge in IGD. Overall, these findings were consistent with our expectations.

## Discussion

The current study structures a moderated mediation model, which reveals the influencing mechanism of PYD on adolescent IGD in mainland China. The results showed that the risk of IGD of individuals with lower levels of PYD was either directly increased or indirectly increased through the higher level of depression. Congruent with the viewpoint of PYD and stage-environment fit theory [2, 20], these negative effects may be rooted in the mismatch between the developmental condition of adolescents and their individual particular requirements. They are not able to interact with the environment and actively integrate their internal and external resources when developmental resources are unable to meet their positive developmental needs, thus leading to emotional and behavioral problems, including depression and IGD.

On the one hand, the findings of this study have theoretical contributions. Based on positive youth development theory, this study blends the internalizing (e.g., depression) and externalizing (e.g., IGD) components of adolescent growth. Instead of solely focusing on a deficit model, we begin to explore important protective factors during this developmental stage, reflecting the change in perspective in adolescent psychology. The present study not only examined the effects of PYD on IGD among Chinese adolescents but also investigated the mediating role of depression and the moderating role of gender, which further deepens and extends previous research findings in IGD. Until now, only a few studies have looked at the role of these variables in the field of quality of life studies in the context of positive youth development paradigms. Therefore, this research adds some additional empirical evidence, particularly in the context of Chinese culture.

On the other hand, the findings of this study also have great practical implications. Our findings highlight the notion that cultivating the approach of PYD as a strategy can foster health and reduce depressive symptoms and IGD among Chinese adolescents. Furthermore, this study can provide a more comprehensive and scientific reference base for the intervention and treatment of adolescent depression and IGD. This demonstrates that schools, educators, and parents should use the PYD approach or PYD programs to facilitate adolescents’ positive development and alleviate their depression and IGD. At the same time, the implementation of screening at the beginning of the academic year would help schools identify adolescents at risk and intervene and prevent them through enhancing positive assets early on [12].

### Positive youth development and internet gaming disorder

This study found that PYD showed a negative predictive effect on adolescent IGD, providing support for Hypothesis 1. In line with prior research [12, 59], PYD attributes aid in the prevention or reduction of teenagers’ addictive behaviors, such as IGD. This finding reveals the protective effect of PYD against IGD in Chinese adolescents. Several studies have reported that some factors of PYD, including parent-child relationships, teacher-student relationships, perceived school climate, school engagement, and teacher autonomy support, all play significant protective roles in IGD [60–64]. Adolescent pupils will face increasing academic pressure and interpersonal relationship problems. If young people do not have sufficient internal assets (e.g. their own cognitive and emotional regulation skills) and external assets (e.g. support and care from parents, teachers, and peers), they are more likely to engage in internet gaming to get a temporary escape.

According to the relation-development-systems model, the development of adolescent adaptability is the result of good interaction between the individual and the context [18]. Adolescents are more likely to be active in their relationships with the environment and less likely to participate in problematic behaviors if they and their ecological surroundings are matched and mutually reinforcing. Youth with strong developmental flexibility will have a similar positive feedback impact on the developmental context, promoting the individual-context link even further. As a result, teachers and parents should provide effective services and support to help students enhance their self-esteem, life satisfaction, and well-being in order to lower the occurrence of IGD [9]. In addition, schools should pay particular attention to the IGD status of young people and build PYD attributes into their regular teaching tasks. For example, develop mental health education programs to improve students’ internal assets; ensure a quality school environment and teaching facilities to provide external support.

### Depression as a mediator

This study revealed that depression mediated the relationship between PYD and IGD among adolescents, proving Hypothesis 2. In other words, having fewer PYD attributes had a facilitative effect on depressive symptoms in adolescents, which in turn exacerbated the negative effects of IGD, similar to the mediating role of depression in previous studies [35, 65]. First, PYD attributes were found to be a negative predictor of depression in our study, congruent with earlier research [35, 66]. Young students with more positive development assets have higher emotional and social abilities and can obtain more support from their society and family [19]. And they are better able to buffer life stress and cope with setbacks, whereas adolescents without these assets are more prone to depression. Furthermore, a longitudinal study also found a strong and stable link between PYD and depression, indicating that promoting the PYD strategy for the treatment of depression in Chinese teenagers is particularly promising [34].

Second, depression positively predicted adolescent IGD, in line with prior findings [37, 38, 62]. According to the cognitive-behavioral model of pathological internet use [67], proposed by Davis, individuals’ nonadaptive behaviors are the result of their predisposed diathesis and life stresses, and depression is one of the distal contributory causes of problematic internet use. Furthermore, the Uses and gratifications (U & G) theory [68] also points out that people use the internet and engage in online gaming to meet their inner needs, and the gratifications from these behaviors alleviate depressive emotions [69]. Generally speaking, depressed teenagers are more prone to turning to the internet for a sense of power and fulfillment in a gamer-avatar connection, as well as to alleviate their despair and develop an addiction to online games [37]. As a consequence, teachers and parents can help young people achieve positive development by fostering internal PYD indicators and providing external PYD assets, both of which are essential for alleviating depression and reducing IGD behaviors. Similarly, this study contributes to the development of positive psychology in a Chinese context, implying that building positive strengths among Chinese adolescents could be a future route for depression intervention [34, 35].

Further, researchers have also found that depression and internet addiction have a complex causal link [46]. One of the motivations for individuals addicted to the internet is to avoid the psychosocial problems that come with depression. They get transient satisfaction from playing online games, but this causes them to lose touch with the real world, resulting in more bad outcomes (including worse grades and alienation from relationships) and exacerbating their loneliness [9, 70, 71]. To break this vicious cycle, we must support the social use of a portion of the internet, on the one hand, and then pay attention to the level of intervention and control over adolescents, on the other hand, which is critical for reducing depression and preventing IGD.

### Gender as a moderator

As expected, the latter half of the mediating impact of depression was moderated by gender in this study, confirming Hypothesis 3. The findings revealed that depressed male adolescents were more likely than female adolescents to be addicted to online gaming, consistent with earlier empirical investigations [7, 8, 10]. In the study of Ko et al. [45], they found that males had a higher tendency than females to report playing online games for the purpose of relieving negative emotions. According to social role theory, gender disparities in social conduct can be explained by social expectations of individual roles [72]. Males are expected to be better at regulating their emotions and addressing problems rationally, while females are expected to be better at expressing their feelings. When men are depressed, they generally hide their emotions. However, the pent-ed emotions require an outlet for release, which leads them to be more likely to turn to an anonymous area of the internet and play online games to satisfy their psychological needs, which makes them more vulnerable to IGD.

Numerous studies have shown that boys gain a greater sense of power and achievement in games and can swiftly improve their self-identity by interacting with like-minded others in online games, which further increases the risk of IGD [7, 45]. Besides, the marketing strategies of games, which are primarily geared at boys, frequently include violence and competition, making them unappealing to girls. Girls are not expected to master the game as well as boys, which is limited by psychosocial issues [73]. Besides, this difference is also reflected in the parenting style; the parents usually give boys less supervision and warmth than girls [74]. Therefore, states and governments should consider implementing special strategies accounting for gender differences, as well as reminding schools, teachers, and parents to provide relevant resources for the positive development of youth, in order to help prevent adolescents with the risk factors from becoming addicted to online gaming. Notably, other studies have indicated that the time difference between males and females playing video games is shrinking, implying that the gender gap in IGD is diminishing and that females will increasingly play online games [75].

### Limitations and prospects

Several limitations should be noted when interpreting the results of this study. First, the current study was conducted using a cross-sectional design, which did not fully infer the causal relation between variables and the mechanism of mediation. Second, although the sample size of this study is relatively large, the data was collected only from two middle schools in Hubei province. Ecological validity is still poor in our research. Third, this study only involved gender and age as control variables. In future studies, researchers should consider more control variables, such as socioeconomic status [59] and family intactness [12]. Fourth, the present findings are based only on self-reported measures, which may result in some response bias, such as individual motives, social desirability, and memory call. According to the findings of Dou and Shek’s study, these measures based on data from important others such as parents, teachers, and peers will add value to the study and reduce some methodological biases [12].

On the basis of the above research results, the following questions can be explored in future research: First of all, although a relative wealth of resources is favorable to the positive development of youth, this positive effect is not merely a cumulative aggregate [76]. So, it will be more advantageous for educators to provide appropriate resources to teenagers if some studies can identify internal and external resources that play a vital role in different contexts, respectively. In addition, PYD attributes include four higher-order structures (including cognitive-behavioral competence, prosocial attributes, positive identity, and general PYD qualities). However, the degree of PYD in this study was determined by averaging the overall score. Maybe these four higher-order dimensions have different predictive effects on IGD [22]. Second, the researchers discovered that a bidirectional relationship existed between Internet addiction and depression. More specifically, depression increases the risk of IGD, and IGD deepens the depressive symptoms in turn, eventually creating a malignant circle [37, 46]. Further research could look into the relationship between depression and IGD, as well as using longitudinal studies to establish causality among considered variables over time. Third, the association between IGD and related factors may differ between males and females. Moreover, examining the factors that play different roles in the underlying mechanisms of IGD in males and females will further enrich the empirical research and provide a foundation for gender differences in IGD, as well as shed new insight into designing different intervention programs for various types of IGD. Finally, earlier IGD research has focused mostly on the impact of the family environment. In recent years, a growing number of studies have found that peer victimization and deviant peer affiliation in the school context might predict depression and IGD [28, 54], reminding educators that the negative peer relationship in PYD is particularly important to consider.

## Conclusion

Based on the perspective of PYD, this study addressed several research gaps in the extant literature regarding the relationship between PYD and IGD among Chinese adolescents. Consistent with our hypotheses, we found that PYD negatively predicted IGD and the mediating role of depression in this association. In addition, gender significantly moderated the second half of the mediated model, with the relationship being stronger for male adolescents. Our findings underline the protective effect of PYD on depressive symptoms and IGD in Chinese adolescents. The present study also suggests that practitioners could provide quality developmental assets or develop more PYD programs aimed to enhance adolescent well-being and healthy Internet game use.

## Supporting information

S1 File Measurements used in present study. (DOCX)

S1 Dataset Dataset used for analyses in present study. (SAV)

## References

[1] Hall GS. Adolescence: Its psychology and its relations to physiology, anthropology, sociology, sex, crime, religion and education. D. Appleton; 1905.

[2] Shek DTL, Dou D, Zhu X, Chai W. Positive youth development: current perspectives. Adolesc Health Med Ther. 2019;10:131–41. doi: 10.2147/AHMT.S179946 . 31572041 PMC6756153

[3] China Internet Network Information Center (CNNIC). The 48th China Statistical Report on Internet Development. 2021;1–75. Available from: http://wlaq.xjtu.edu.cn/48hlbg.pdf.

[4] Xi W, Hu YZ. Internet Gaming Disorder in Adolescents: Review and Prospect. Chinese Journal of Applied Psychology. 2022;28(01):3–19.

[5] Young KS. Internet Addiction. American Behavioral Scientist. 2004;48(4):402–15. 10.1177/0002764204270278.

[6] King DL, Potenza MN. Not Playing Around: Gaming Disorder in the International Classification of Diseases (ICD-11). J Adolesc Health. 2019;64(1):5–7. doi: 10.1016/j.jadohealth.2018.10.010 . 30579437

[7] Chiu SI, Lee JZ, Huang DH. Video Game Addiction in Children and Teenagers in Taiwan. CyberPsychology & Behavior. 2004;7(5):571–81. doi: 10.1089/cpb.2004.7.571 15667052

[8] Griffiths MD, Davies MNO, Chappell D. Online computer gaming: a comparison of adolescent and adult gamers. J Adolesc. 2004;27(1):87–96. doi: 10.1016/j.adolescence.2003.10.007 . 15013262

[9] Lemmens JS, Valkenburg PM, Peter J. Psychosocial causes and consequences of pathological gaming. Computers in Human Behavior. 2011;27(1):144–52. 10.1016/j.chb.2010.07.015.

[10] Toker S, Baturay MH. Antecedents and consequences of game addiction. Computers in Human Behavior. 2016;55:668–79. 10.1016/j.chb.2015.10.002.

[11] Chen R, Chen X, Zhao G. Effect of positive youth development on problem behavior: Mediated by well-being. China Journal of Health Psychology. 2020;28(12):1847–53. 10.13342/j.cnki.cjhp.2020.12.021.

[12] Dou D, Shek DTL. Concurrent and Longitudinal Relationships between Positive Youth Development Attributes and Adolescent Internet Addiction Symptoms in Chinese Mainland High School Students. Int J Environ Res Public Health. 2021;18(4). doi: 10.3390/ijerph18041937 . 33671277 PMC7922687

[13] Yu L, Shek DTL. Positive youth development attributes and parenting as protective factors against adolescent social networking addiction in Hong Kong. Frontiers in Pediatrics. 2021; 9:649232. doi: 10.3389/fped.2021.649232 33816410 PMC8012543

[14] Abo-Zena MM, Rana M. Ecological Perspectives on Religion and Positive Youth Development. Religions. 2020;11(8). 10.3390/rel11080406.

[15] Guo H-j, Liu F, Liu W, Lin X-y, Lin D-h. Positive Youth Development: Theory, Practice, and Prospective. Journal of Beijing Normal University (Social Sciences). 2017;(06):5–13.

[16] Gestsdottir S, Urban JB, Bowers EP, Lerner JV, Lerner RM. Intentional self-regulation, ecological assets, and thriving in adolescence: a developmental systems model. New Dir Child Adolesc Dev. 2011;2011(133):61–76. doi: 10.1002/cd.304 . 21898899

[17] Catalano RF, Berglund ML, Ryan JAM, Lonczak HS, Hawkins JD. Positive Youth Development in the United States: Research Findings on Evaluations of Positive Youth Development Programs. The annals of the American Academy of Political and Social Science. 2004;591(1):98–124. 10.1177/0002716203260102.

[18] Lerner RM, Lerner JV, P. Bowers EP, Geldhof GJ. Positive Youth Development and Relational-Developmental-Systems. Handbook of Child Psychology and Developmental Science. 2015:1–45. 10.1002/9781118963418.childpsy11.

[19] Atkiss K, Moyer M, Desai M, Roland M. Positive Youth Development. American Journal of Health Education. 2013;42(3):171–80. 10.1080/19325037.2011.10599184.

[20] Gutman LM, Eccles JS. Stage-environment fit during adolescence: trajectories of family relations and adolescent outcomes. Dev Psychol. 2007;43(2):522–37. doi: 10.1037/0012-1649.43.2.522 . 17352557

[21] Ma CMS. The Relationship Between Social Support and Life Satisfaction Among Chinese and Ethnic Minority Adolescents in Hong Kong: the Mediating Role of Positive Youth Development. Child Indicators Research. 2019;13(2):659–79. 10.1007/s12187-019-09638-2.

[22] Zhu X, Shek DTL. Predictive Effect of Positive Youth Development Attributes on Delinquency Among Adolescents in Mainland China. Front Psychol. 2020;11:615900. doi: 10.3389/fpsyg.2020.615900 . 33381073 PMC7768043

[23] Sun RCF, Shek DTL. Positive Youth Development, Life Satisfaction and Problem Behaviour Among Chinese Adolescents in Hong Kong: A Replication. Social Indicators Research. 2012;105(3):541–59. doi: 10.1007/s11205-011-9786-9 22247583 PMC3249555

[24] Sun RCF, Shek DTL. Longitudinal Influences of Positive Youth Development and Life Satisfaction on Problem Behaviour among Adolescents in Hong Kong. Social Indicators Research. 2012;114(3):1171–97. 10.1007/s11205-012-0196-4. PMC3249555 22247583

[25] Shek DTL, Siu AMH, Lee TY, Cheung CK, Chung R. Effectiveness of the Tier 1 Program of Project P.A.T.H.S.: objective outcome evaluation based on a randomized group trial. ScientificWorldJournal. 2008;8:4–12. doi: 10.1100/tsw.2008.16 . 18246283 PMC5848723

[26] Zhu X, Shek DTL. Subjective Outcome Evaluation of a Positive Youth Development Program in Mainland China. Research on Social Work Practice. 2020;31(3):285–97. 10.1177/1049731520980802.

[27] Zhang W. Adolescent developmental psychology. Shandong: Shandong people’s publishing house; 2002.

[28] Liu J, Bullock A, Coplan RJ, Chen X, Li D, Zhou Y. Developmental cascade models linking peer victimization, depression, and academic achievement in Chinese children. Br J Dev Psychol. 2017;36(1):47–63. doi: 10.1111/bjdp.12212 . 28975639

[29] Purdon C. Review of the prevention of anxiety and depression: Theory, research and practice. Canadian Psychology/Psychologie Canadienne. 2004;45(3):249–50. 10.1037/h0088068.

[30] Zubrick SR, Hafekost J, Johnson SE, Sawyer MG, Patton G, Lawrence D. The continuity and duration of depression and its relationship to non-suicidal self-harm and suicidal ideation and behavior in adolescents 12–17. J Affect Disord. 2017;220:49–56. doi: 10.1016/j.jad.2017.05.050 . 28595098

[31] McLeod GFH, Horwood LJ, Fergusson DM. Adolescent depression, adult mental health and psychosocial outcomes at 30 and 35 years. Psychol Med. 2016;46(7):1401–12. doi: 10.1017/S0033291715002950 . 26818194

[32] Chang S, Zhang W. The Developmental Assets Framework of Positive Human Development: An Important Approach and Field in Positive Youth Development Study. Advances in Psychological Science. 2013;21(01):86–95.

[33] Milot Travers AS, Mahalik JR. Positive youth development as a protective factor for adolescents at risk for depression and alcohol use. Applied Developmental Science. 2019;25(4):322–31. 10.1080/10888691.2019.1634569.

[34] Chi X, Liu X, Huang Q, Cui X, Lin L. The Relationship between Positive Youth Development and Depressive Symptoms among Chinese Early Adolescents: A Three-Year Cross-Lagged Analysis. Int J Environ Res Public Health. 2020;17(17). doi: 10.3390/ijerph17176404 . 32887499 PMC7503901

[35] Zhou Z, Shek DTL, Zhu X, Dou D. Positive Youth Development and Adolescent Depression: A Longitudinal Study Based on Mainland Chinese High School Students. International Journal of Environmental Research and Public Health. 2020;17(12):4457. doi: 10.3390/ijerph17124457 32575856 PMC7344806

[36] Carli V, Durkee T, Wasserman D, Hadlaczky G, Despalins R, Kramarz E, et al. The association between pathological internet use and comorbid psychopathology: a systematic review. Psychopathology. 2013;46(1):1–13. doi: 10.1159/000337971 . 22854219

[37] Burleigh TL, Stavropoulos V, Liew LWL, Adams BLM, Griffiths MD. Depression, Internet Gaming Disorder, and the Moderating Effect of the Gamer-Avatar Relationship: an Exploratory Longitudinal Study. International Journal of Mental Health and Addiction. 2017;16(1):102–24. 10.1007/s11469-017-9806-3.

[38] Ryu H, Lee JY, Choi A, Park S, Kim DJ, Choi JS. The Relationship between Impulsivity and Internet Gaming Disorder in Young Adults: Mediating Effects of Interpersonal Relationships and Depression. International Journal of Environmental Research and Public Health. 2018;15(3):1–11. doi: 10.3390/ijerph15030458 29509708 PMC5877003

[39] Stavropoulos V, Gentile D, Motti-Stefanidi F. A multilevel longitudinal study of adolescent Internet addiction: The role of obsessive–compulsive symptoms and classroom openness to experience. European Journal of Developmental Psychology. 2015;13(1):99–114. 10.1080/17405629.2015.1066670.

[40] Gao W, Chen Z. A Study on Psychopathology and Psychotherapy of Internet Addiction. Advances in Psychological Science. 2006;14(04):596–603.

[41] Wei H, Zhou Z-k, Tian Y, Bao N. Online Game Addiction: Effects and Mechanisms of Flow Experience. Psychological Development and Education. 2012;28(06):651–7.

[42] Wang L. A Brief Theoretical and Empirical Review of Gender Identity Research. Journal of Taiyuan University (Natural Science Edition). 2016;34(04):22–6. 10.14152/j.cnki.2096-191X.2016.04.008.

[43] Carver PR, Yunger JL, Perry DG. Gender identity and Adjustment in Middle Childhood. Sex Roles. 2003;49(3/4):95–109.

[44] Lin Y, Liu Q, Yu S, Zhou Z. The Relationship between Parents Neglect and Online Gaming Addiction among Adolescents: The Mediating Role of Hope and Gender Difference. Psychological Development and Education. 2021;37(1):109–19. 10.16187/j.cnki.issn1001-4918.2021.01.14.

[45] Ko CH, Yen JY, Chen CC, Chen SH, Yen CF. Gender differences and related factors affecting online gaming addiction among Taiwanese adolescents. J Nerv Ment Dis. 2005;193(4):273–7. doi: 10.1097/01.nmd.0000158373.85150.57 . 15805824

[46] Liang L, Zhou D, Yuan C, Shao A, Bian Y. Gender differences in the relationship between internet addiction and depression: A cross-lagged study in Chinese adolescents. Computers in Human Behavior. 2016;63:463–70. 10.1016/j.chb.2016.04.043.

[47] Shek DTL, Siu AMH, Lee TY. The Chinese Positive Youth Development Scale. Research on Social Work Practice. 2007;17(3):380–91. 10.1177/1049731506296196.

[48] Chi X, Liu X, Guo T, Chen X. Internet Addiction and Depression in Chinese Adolescents: A Moderated Mediation Model. Front Psychiatry. 2019;10:816. doi: 10.3389/fpsyt.2019.00816 . 31798471 PMC6865207

[49] Radloff LS. The CES-D Scale: A Self-Report Depression Scale for Research in the General Population. Applied Psychological Measurement. 1977;1(3):385–401. 10.1177/014662167700100306

[50] Wang X, Wang X, Ma H. Handbook of Mental Health Rating Scale (updated version). Beijing: Chinese Mental Health Journal; 1999.

[51] Chen Z-y, Yang X-d, Li X-y. Psychometric Features of CES-D in Chinese Adolescents. Chinese Journal of Clinical Psychology. 2009;17(04):443–5,8.

[52] Gentile D. Pathological Video-Game Use Among Youth Ages 8 to 18. Psychological Science. 2009;20(5):594–602. 10.1111/j.1467-9280.2009.02340.x. 19476590

[53] Yu C, Tang C, Lin Z, Zhang Q. The Interplay between Multilevel Individual and Environmental Factors Acting on the Internet Gaming Disorder in Adolescents: Based on the Latent Profile Analysis. Educational Measurement and Evaluation. 2017;(06):33–44, 51. 10.16518/j.cnki.emae.2017.06.006.

[54] Li M-m, Gan X, Jin X. Marital Conflicts and Internet Gaming Disorder in Adolescents: Multiple Mediations of Deviant Peer Affiliation and Neuroticism. Chinese Journal of Clinical Psychology. 2020;28(02):354–8. 10.16128/j.cnki.1005-3611.2020.02.028.

[55] Podsakoff PM, MacKenzie SB, Lee JY, Podsakoff NP. Common method biases in behavioral research: a critical review of the literature and recommended remedies. J Appl Psychol. 2003;88(5):879–903. doi: 10.1037/0021-9010.88.5.879 . 14516251

[56] Zhou H, Long L. Statistical Remedies for Common Method Biases. Advances in Psychological Science. 2004;(06):942–50.

[57] Preacher KJ, Hayes AF. Asymptotic and resampling strategies for assessing and comparing indirect effects in multiple mediator models. Behav Res Methods. 2008;40(3):879–91. doi: 10.3758/brm.40.3.879 . 18697684

[58] Hayes AF. Introduction to mediation, moderation, and conditional process analysis: a regression-based approach. New York: Guilford Press; 2013.

[59] Phelps E, Balsano AB, Fay K, Peltz JS, Zimmerman SM, Lerner RM, et al. Nuances in early adolescent developmental trajectories of positive and problematic/risk behaviors: findings from the 4-H study of positive youth development. Child Adolesc Psychiatr Clin N Am. 2007;16(2):473–96. doi: 10.1016/j.chc.2006.11.006 . 17349519

[60] Bao X, Zhang W, Yu C, Zhu J, Bao Z, Jiang Y, et al. Perceived School Climate and Internet Gaming Disorder Among Junior Middle School Students: The Mediating Role of Academic Self-efficacy and The Moderating Role of Parental Academic Involvement. Psychological Development and Education. 2016;32(03):358–68. 10.16187/j.cnki.issn1001-4918.2016.03.13.

[61] Tian Y, Yu C, Lin S, Ye S, Zhang X, Liu Y, et al. Parental Corporal Punishment, School Engagement and Internet Gaming Addiction among Adolescents: Parent-Adolescent Relationship as a Moderator. Psychological Development and Education. 2018;34(04):461–71. 10.16187/j.cnki.issn1001-4918.2018.04.10.

[62] Wang J, Yu C, Liu S, Li W. Family Cohesion, Depression and Adolescent Internet Gaming Disorder: The Moderating Role of COMT Gene rs737866 Polymorphism. Journal of Lanzhou University (Social Sciences). 2020;48(04):143–9. 10.13885/j.issn.1000-2804.2020.04.016.

[63] Yu C, Li X, Zhang W. Predicting adolescent problematic online game use from teacher autonomy support, basic psychological needs satisfaction, and school engagement: a 2-year longitudinal study. Cyberpsychol Behav Soc Netw. 2015;18(4):228–33. doi: 10.1089/cyber.2014.0385 . 25803769

[64] Zhang X, Ye S, Yu C, Lu H. Teacher- Student Relationship and Internet Addiction Disorder of Teenagers: the Mediating Effect of School Participation and Regulation Effect of Future Orientation. Educational Measurement and Evaluation. 2018;(02):58–64. 10.16518/j.cnki.emae.2018.02.010.

[65] Yen JY, Chou WP, Lin HC, Wu HC, Tsai WX, Ko CH. Roles of Hostility and Depression in the Association between the MAOA Gene Polymorphism and Internet Gaming Disorder. International Journal of Environmental Research and Public Health. 2021;18(13):6910. doi: 10.3390/ijerph18136910 34199135 PMC8297287

[66] Liu X, Wang Q, Chi X, Qi D. Family function and Depression in Adolescents: A Moderated Mediation Model. Chinese Journal of Clinical Psychology. 2020;28:688–93,772. 10.16128/j.cnki.1005-3611.2020.04.008.

[67] Davis RA. A cognitive-behavioral model of pathological Internet use. Computers in Human Behavior. 2001;17:187–95. 10.1016/s0747-5632(00)00041-8.

[68] Roy SK. Internet uses and gratifications: A survey in the Indian context. Computers in Human Behavior. 2009;25(4):878–86. 10.1016/j.chb.2009.03.002.

[69] Stafford TF, Stafford MR, Schkade LL. Determining Uses and Gratifications for the Internet. Decision Sciences. 2004;35(2):259–88. 10.1111/j.00117315.2004.02524.x.

[70] Kim J, LaRose R, Peng W. Loneliness as the Cause and the Effect of Problematic Internet Use: The Relationship between Internet use and Psychological Well-being. CyberPsychology & Behavior. 2009;12(4):451–5. doi: 10.1089/cpb.2008.0327 19514821

[71] Yao MZ, Zhong Z-j. Loneliness, social contacts and Internet addiction: A cross-lagged panel study. Computers in Human Behavior. 2014;30:164–70. 10.1016/j.chb.2013.08.007.

[72] Xie J, Hong Y, Tang H, Shen C. Effects Mechanism of Gender on Work-to-family Conflict: An Empirical Study Based on the Social Role Theory. Journal of Psychological Science. 2015;38(1):191–5. 10.16719/j.cnki.1671-6981.2015.01.024.

[73] Cruea M, Park S-Y. Gender Disparity in Video Game Usage: A Third-Person Perception-Based Explanation. Media Psychology. 2012;15(1):44–67. 10.1080/15213269.2011.648861.

[74] Liu Y, Li X. Influence of parenting style and gender disparity on adolescent problem behaviors. China Journal of Health Psychology. 2015;31(12):1655–7.

[75] Homer BD, Hayward EO, Frye J, Plass JL. Gender and player characteristics in video game play of preadolescents. Computers in Human Behavior. 2012;28(5):1782–9. doi: 10.1016/j.chb.2012.04.018

[76] Cai X, Lin D. School transition during adolescence: Turning crisis into opportunity. Advances in Psychological Science. 2021;29(05):864–74.

