# Supplementary material for: The relationship between positive youth development and internet gaming disorder in Chinese adolescents: A moderated mediation model
Source: PLoS One. 2022 Nov 3;17(11):e0276174. doi: 10.1371/journal.pone.0276174 (PMC9632815; doi:10.1371/journal.pone.0276174)
Supplement: S1 File — (DOCX) [file pone.0276174.s001.docx]

**Youth Learning Life Questionnaire**

**Dear Students:**

Hello! First of all, I am sorry for taking up your precious time! But I really need your support! This is a questionnaire about the academic lives of young people, and your careful and honest answers are the basis and key to the success of this survey. The information contained in this questionnaire will be kept strictly confidential and will only be used for scientific research. The questionnaire will take approximately 20-30 minutes to complete. To ensure the validity of the survey data, please read the questions carefully and fill them in truthfully, without omission. We look forward to your enthusiastic cooperation. Thank you!

**The following questions are used to get a general idea of your situation. Please read each question and the corresponding answer carefully. Do not spend too much time on any one question. Choose the answer that best matches your current situation and tick the appropriate option.**

1. Gender: ①Male ②Female

2. Age:

3. Grade:

①Junior High School Year 1 ②Junior High School Year 2

③Junior High School Year 3 ④High School Year 1 ⑤High School Year 2 ⑥High School Year 3

**Part I**

**Please indicate the extent to which each of the following statements reflects the situation in your academic life by ticking the number that corresponds to the appropriate extent according to your own circumstances (1 = Disagree Strongly, 2 = Disagree Somewhat, 3 = Disagree A Little, 4 = Agree A Little, 5 = Agree Somewhat, 6 = Agree Strongly).**

| This questionnaire consists of 90 questions with 6 options for each question. Please be careful not to miss any questions. Only one answer is allowed for each question, otherwise the whole questionnaire will be invalidated. | Disagree Strongly | Disagree Somewhat | Disagree A  Little | Agree  A  Little | Agree  Some-what | Agree  Strong-ly |
| --- | --- | --- | --- | --- | --- | --- |
| Ⅰ： | | | | | | |
| 1. When I need help, I trust my parents to help me. | 1 | 2 | 3 | 4 | 5 | 6 |
| 2. When I need help, I trust my friends to help me. | 1 | 2 | 3 | 4 | 5 | 6 |
| 3. I have a good sense of self. | 1 | 2 | 3 | 4 | 5 | 6 |
| 4. When I need help, I trust my teacher to help me. | 1 | 2 | 3 | 4 | 5 | 6 |
| 5. I love my teachers and classmates. | 1 | 2 | 3 | 4 | 5 | 6 |
| 6. I have a lot of helpful friends in my life. | 1 | 2 | 3 | 4 | 5 | 6 |
| 7. My parents care about me. | 1 | 2 | 3 | 4 | 5 | 6 |
| Ⅱ： | | | | | | |
| 8. When I face difficulties, I do not give up easily. | 1 | 2 | 3 | 4 | 5 | 6 |
| 9. When I face adversity, I remain optimistic. | 1 | 2 | 3 | 4 | 5 | 6 |
| 10. I believe that where there is a will, there is a way. | 1 | 2 | 3 | 4 | 5 | 6 |
| 11. I believe that those who can endure suffering have exceptional personal qualities. | 1 | 2 | 3 | 4 | 5 | 6 |
| 12. My belief is that even if tomorrow is bad, I will face it well. | 1 | 2 | 3 | 4 | 5 | 6 |
| 13. I believe that life's problems can be solved. | 1 | 2 | 3 | 4 | 5 | 6 |
| Ⅲ： | | | | | | |

|  | Disagree Strongly | Disagree Somewhat | | Disagree A  Little | Agree  A  Little | Agree  Some-what | Agree  Strong-ly |
| --- | --- | --- | --- | --- | --- | --- | --- |
| 14. I talk positively to strangers. | 1 | 2 | | 3 | 4 | 5 | 6 |
| 15. I know how to communicate with others. | 1 | 2 | | 3 | 4 | 5 | 6 |
| 16. I understand the rules and expectations for interfacing with others. | 1 | 2 | | 3 | 4 | 5 | 6 |
| 17. I can interact with others in a harmonious way. | 1 | 2 | | 3 | 4 | 5 | 6 |
| 18. I like to participate in social activities. | 1 | 2 | | 3 | 4 | 5 | 6 |
| 19. I know how to distinguish between good friends and bad friends. | 1 | 2 | | 3 | 4 | 5 | 6 |
| 20. I know how to listen to people. | 1 | 2 | | 3 | 4 | 5 | 6 |
| Ⅳ： | | | | | | | |
| 21. I am a pleasant person. | 1 | 2 | | 3 | 4 | 5 | 6 |
| 22. I can express my emotions appropriately when I am unhappy. | 1 | 2 | | 3 | 4 | 5 | 6 |
| 23. When I am angry, I can describe my feelings rationally. | 1 | 2 | | 3 | 4 | 5 | 6 |
| 24. I can manage my emotions when I have conflicts with others. | 1 | 2 | | 3 | 4 | 5 | 6 |
| 25. I can see the world from someone else's point of view. | 1 | 2 | | 3 | 4 | 5 | 6 |
| 26 I will let people know my emotions. | 1 | 2 | | 3 | 4 | 5 | 6 |
| Ⅴ： | | | | | | | |
| 27. I believe that there is a solution to any problem. | 1 | 2 | 3 | | 4 | 5 | 6 |
| 28. I know how to see things from different perspectives. | 1 | 2 | 3 | | 4 | 5 | 6 |
| 29. I will try to solve my problems in new ways. | 1 | 2 | 3 | | 4 | 5 | 6 |
| 30. I know how to identify the cause of a problem and its solution. | 1 | 2 | 3 | | 4 | 5 | 6 |
| 31. I know how to make plans to achieve my goals. | 1 | 2 | 3 | | 4 | 5 | 6 |
| 32. I can distinguish between the good and bad aspects of things. | 1 | 2 | 3 | | 4 | 5 | 6 |
| Ⅵ： | | | | | | | |
| 33. I know how to refuse an unfair request. | 1 | 2 | 3 | | 4 | 5 | 6 |
| 34. I know how to make careful decisions when I am persuaded to do so. | 1 | 2 | 3 | | 4 | 5 | 6 |
| 35. I can face criticism with an open mind. | 1 | 2 | 3 | | 4 | 5 | 6 |
| 36. I am able to express a different point of view from others. | 1 | 2 | 3 | | 4 | 5 | 6 |
| 37. When I do something wrong, I apologize to the person concerned. | 1 | 2 | 3 | | 4 | 5 | 6 |
| 38. I spend more time avoiding problems than solving them. | 1 | 2 | 3 | | 4 | 5 | 6 |
| Ⅶ： | | | | | | | |
| 39. I like to share my things with others. | 1 | 2 | 3 | | 4 | 5 | 6 |
| 40. I have high moral expectations of my behavior. | 1 | 2 | 3 | | 4 | 5 | 6 |
| 41. It is easy for me to forgive those who offend me. | 1 | 2 | 3 | | 4 | 5 | 6 |
| 42. I will not do anything that is unfair to others. | 1 | 2 | 3 | | 4 | 5 | 6 |
| 43. I will keep my word. | 1 | 2 | 3 | | 4 | 5 | 6 |
|  | Disagree Strongly | Disagree Somewhat | Disagree A  Little | | Agree  A  Little | Agree  Some-what | Agree  Strong-ly |
| 44. I am in the habit of self-evaluation. | 1 | 2 | 3 | | 4 | 5 | 6 |
| Ⅷ： | | | | | | | |
| 45. I am able to make informed choices. | 1 | 2 | 3 | | 4 | 5 | 6 |
| 46. I have confidence in my decision. | 1 | 2 | 3 | | 4 | 5 | 6 |
| 47. I don't change my mind easily when I make a decision. | 1 | 2 | 3 | | 4 | 5 | 6 |
| 48. I can concentrate on the task. | 1 | 2 | 3 | | 4 | 5 | 6 |
| 49. I have the freedom to do what I want to do. | 1 | 2 | 3 | | 4 | 5 | 6 |
| Ⅸ： | | | | | | | |
| 50. I have little control over what happens in my life. | 1 | 2 | 3 | | 4 | 5 | 6 |
| 51. I do not have any solutions to some of the problems I am facing. | 1 | 2 | 3 | | 4 | 5 | 6 |
| 52. I can't do much to change things in my life. | 1 | 2 | 3 | | 4 | 5 | 6 |
| 53. I feel helpless when I encounter difficulties in life. | 1 | 2 | 3 | | 4 | 5 | 6 |
| 54. I feel that my life depends on other people and fate. | 1 | 2 | 3 | | 4 | 5 | 6 |
| 55. I believe that most of the things that happen in my life are decided by me. | 1 | 2 | 3 | | 4 | 5 | 6 |
| 56. I can accomplish almost everything I am determined to do. | 1 | 2 | 3 | | 4 | 5 | 6 |
| Ⅹ： | | | | | | | |
| 57. I usually feel bored with life rather than enjoy it very much. | 1 | 2 | 3 | | 4 | 5 | 6 |
| 58. For me, life is very dull rather than exciting. | 1 | 2 | 3 | | 4 | 5 | 6 |
| 59. I would rather not exist at all than choose the life I have now. | 1 | 2 | 3 | | 4 | 5 | 6 |
| 60. My goal in life is to be unprogressive rather than full of achievement. | 1 | 2 | 3 | | 4 | 5 | 6 |
| 61. My life is empty, not full of excitement. | 1 | 2 | 3 | | 4 | 5 | 6 |
| 62. I feel confused rather than feeling meaningful in my relationship with the world. | 1 | 2 | 3 | | 4 | 5 | 6 |
| 63. I see suicide as a solution to life, rather than not having considered it. | 1 | 2 | 3 | | 4 | 5 | 6 |
| Ⅺ： | | | | | | | |
| 64. I am confident that I can solve the problems of my future. | 1 | 2 | 3 | | 4 | 5 | 6 |
| 65. I am confident of being accepted to university. | 1 | 2 | 3 | | 4 | 5 | 6 |
| 66. I am confident that I will be a useful person when I grow up. | 1 | 2 | 3 | | 4 | 5 | 6 |
| 67. I don't expect to get what I want. | 1 | 2 | 3 | | 4 | 5 | 6 |
| 68. I can see a poor future for me. | 1 | 2 | 3 | | 4 | 5 | 6 |
|  | Disagree Strongly | Disagree Somewhat | Disagree A  Little | | Agree  A  Little | Agree  Some-what | Agree  Strong-ly |
| 69. It is impossible for me to achieve satisfaction in the future. | 1 | 2 | 3 | | 4 | 5 | 6 |
| 70. It is likely that I will not get what I want in the future. | 1 | 2 | 3 | | 4 | 5 | 6 |
| Ⅻ： | | | | | | | |
| 71. I can do things as well as anyone else. | 1 | 2 | 3 | | 4 | 5 | 6 |
| 72. I am satisfied with my performance compared to my classmates. | 1 | 2 | 3 | | 4 | 5 | 6 |
| 73. I am happy with my body and my appearance. | 1 | 2 | 3 | | 4 | 5 | 6 |
| 74. I feel that I am welcomed by others. | 1 | 2 | 3 | | 4 | 5 | 6 |
| 75. I am a confident person. | 1 | 2 | 3 | | 4 | 5 | 6 |
| 76. I am a dutiful person. | 1 | 2 | 3 | | 4 | 5 | 6 |
| 77. I know my strengths and weaknesses. | 1 | 2 | 3 | | 4 | 5 | 6 |
| ⅰ： | | | | | | | |
| 78. The University encourages students to participate in voluntary service. | 1 | 2 | 3 | | 4 | 5 | 6 |
| 79. I am aware of the avenues for volunteering. | 1 | 2 | 3 | | 4 | 5 | 6 |
| 80. Our school encourages students to help each other. | 1 | 2 | 3 | | 4 | 5 | 6 |
| 81. My classmates were encouraged to share with each other. | 1 | 2 | 3 | | 4 | 5 | 6 |
| 82. I will do my best to contribute to my school or community. | 1 | 2 | 3 | | 4 | 5 | 6 |
| ⅱ： | | | | | | | |
| 83. I care for the less fortunate in society. | 1 | 2 | 3 | | 4 | 5 | 6 |
| 84. If given the opportunity, I would do voluntary work. | 1 | 2 | 3 | | 4 | 5 | 6 |
| 85. I agree that everyone should be bound by the law. | 1 | 2 | 3 | | 4 | 5 | 6 |
| 86. I am happy to abide by the school rules. | 1 | 2 | 3 | | 4 | 5 | 6 |
| ⅲ： | | | | | | | |
| 87. My teacher praises me when I complete my tasks. | 1 | 2 | 3 | | 4 | 5 | 6 |
| 88. When I help others, my classmates will recognize my actions. | 1 | 2 | 3 | | 4 | 5 | 6 |
| 89. The teacher is concerned about whether I have completed my tasks. | 1 | 2 | 3 | | 4 | 5 | 6 |
| 90. The University has clear criteria for rewards and sanctions. | 1 | 2 | 3 | | 4 | 5 | 6 |

**Part II**

**Please indicate the extent to which each of the following statements reflects how often you play, and tick the number that corresponds to the corresponding frequency according to your actual situation (1 = never, 2 = sometimes, 3 = often).**

| **This questionnaire consists of 11 questions with 3 options for each question. Please be careful not to miss any questions and only one answer is allowed for each question, otherwise the whole questionnaire will be invalidated.** | **Never** | **Sometimes** | **Frequently** |
| --- | --- | --- | --- |
| 1. Have you ever been overly preoccupied with online games (for example, not going online and then thinking about a scene from an online game you just played or planning to return to it the next time? | 1 | 2 | 3 |
| 2. Have you had to spend more and more time on online games to get satisfaction? | 1 | 2 | 3 |
| 3. Have you tried and failed to reduce the number of times or the amount of time you spend playing online games? | 1 | 2 | 3 |
| 4. Have you become agitated or irritable when you try to cut down or stop playing online games? | 1 | 2 | 3 |
| 5. Have you ever played online games to avoid problems or bad feelings? | 1 | 2 | 3 |
| 6. Have you ever lied to your parents or friends about how often you play online games? | 1 | 2 | 3 |
| 7. Have you ever stolen electronic equipment from a shop or a friend, or have you ever stolen money to play an online game? | 1 | 2 | 3 |
| 8. Have you ever stolen an electronic device from a shop or a friend, or have you ever stolen money to play an online game? | 1 | 2 | 3 |
| 9. Have you ever skipped homework to have more time to play online games? | 1 | 2 | 3 |
| 10. Have you ever achieved poor academic results or performed poorly in exams because you spent too much time on online games? | 1 | 2 | 3 |
| 11. Have you ever needed friends or family to give you extra money because you spent too much money on online gaming equipment, software, or games/networking? | 1 | 2 | 3 |

**Part III**

**The following is a description of how you may be feeling or have felt recently. Please tick the appropriate box according to how you have actually felt in the past week. 1 = occasionally or not at all (less than 1 day), 2 = sometimes (1 to 2 days), 3 = often or half the time (3 to 4 days), 4 = most of the time or constantly (5 to 7 days).**

| **This questionnaire consists of 20 questions with four options for each question. Please be careful not to miss any questions. Only one answer is allowed for each question, otherwise the whole questionnaire will be invalidated.** | **occasionally or not at all** | **sometimes** | **often or half the time** | **most of the time or constantly** |
| --- | --- | --- | --- | --- |
| 1. I am troubled by little things. | 1 | 2 | 3 | 4 |
| 2. I don't really want to eat and my appetite is not good. | 1 | 2 | 3 | 4 |
| 3. Even if my family or friends help me, I still can't get rid of the bitterness in my heart. | 1 | 2 | 3 | 4 |
| 4. I feel I am as good as anyone else. | 1 | 2 | 3 | 4 |
| 5. It is difficult for me to concentrate on what I am doing. | 1 | 2 | 3 | 4 |
| 6. I feel emotionally depressed. | 1 | 2 | 3 | 4 |
| 7. I feel overwhelmed by everything I do. | 1 | 2 | 3 | 4 |
| 8. I think there is hope for the future. | 1 | 2 | 3 | 4 |
| 9. I think my life is worthless. | 1 | 2 | 3 | 4 |
| 10. I feel frightened and scared. | 1 | 2 | 3 | 4 |
| 11. My sleep situation is not good. | 1 | 2 | 3 | 4 |
| 12. I am happy. | 1 | 2 | 3 | 4 |
| 13. I have been talking less than usual in the last week. | 1 | 2 | 3 | 4 |
| 14. I feel alone. | 1 | 2 | 3 | 4 |
| 15. I don't think people are very nice to me. | 1 | 2 | 3 | 4 |
| 16. I think life is interesting. | 1 | 2 | 3 | 4 |
| 17. I used to cry. | 1 | 2 | 3 | 4 |
| 18. I feel sad. | 1 | 2 | 3 | 4 |
| 19. I don't think people like me. | 1 | 2 | 3 | 4 |
| 20. I feel unable to continue with my daily life. | 1 | 2 | 3 | 4 |

**Thank you for your participation!**
